# Scientifc Evidence on the Supportive Cancer Care with Chinese Medicine

**DOI:** 10.3779/j.issn.1009-3419.2010.03.01

**Published:** 2010-03-20

**Authors:** CS CHO William

**Affiliations:** Department of Clinical Oncology, Queen Elizabeth Hospital, Hong Kong SAR 香港特别行政区，伊利沙伯医院，临床肿瘤科

**Keywords:** Acupuncture, Cancer, Chinese medicine, Meta-analysis, Neoplasms, Qigong, Randomized controlled trial, Supportive care, 针灸, 癌症, 中医药, 荟萃分析, 肿瘤, 气功, 随机对照试验, 支持性治疗

## Abstract

Complementary and alternative medicine has been increasingly utilized by cancer patients in developed countries. Among the various forms of complementary and alternative medicine, Traditional Chinese Medicine is one of the few that has a well constructed theoretical framework and established treatment approaches for diseases including cancer. Recent research has revealed growing evidence suggesting that Traditional Chinese Medicine is effective in the supportive care of cancer patients during and after major conventional cancer treatments. This paper succinctly summarizes some published clinical evidence and meta-analyses which support the usage of various Traditional Chinese Medicine treatment strategies including Chinese herbal medicine, acupuncture and Qigong in supportive cancer care.

## Abstract (zh)

辅助与替代疗法已被越来越多用于已发展国家的癌症患者。在各种另类疗法中，只有少数像中医药般既具坚实理论、又有完备的治病方法，包括对癌症的治疗。近年，许多证据表明中医药是有效的癌症支持性治疗，可在常规治疗的治疗期间和治疗后应用。本文特归纳一些已发表的临床报告和荟萃分析，综述中医药治疗方案对癌症支持性治疗的证据，其中包括中药材、针灸和气功治疗。

## Introduction

Up to 80% of cancer patients in the Western countries have utilized some forms of complementary and alternative medicine (CAM) to support their conventional cancer therapies^[1, 2]^. In 2005, surveys revealed that 44.6% of cancer patients in Japan and 35.9% of cancer patients in 14 European countries used CAM therapies^[3, 4]^. These results are consistent with similar surveys conducted in other regions of the world^[5]^.

Among the various forms of CAM, Traditional Chinese Medicine (TCM) is one of the few that has a well constructed theoretical framework and established treatment approaches for diseases including cancer. The use of TCM for the management of cancers can be traced back to Shang Dynasty of 3 500 year ago. Over the centuries, various TCM therapeutic interventions such as herbal medicine, acupuncture, moxibustion and Qigong have been developed and employed in cancer treatment^[6]^. Although there has been a substantial increase in randomized controlled trials (RCTs) over the past two decades, conclusive evidence to support the use of these interventions is still generally lacking. In this paper, some lines of scientific evidence and systematic reviews on these captioned areas will be discussed.

## Supportive cancer care with Chinese herbal medicine

### Enhancing immune function

The development of a clinically apparent cancer is due, in part, to the failure of the immune system to adequately recognize and dispose of the initial malignant cells. Thus, cancer may be regarded as an immune system failure. The ideal oncologic treatment would not only attack the cancer cells directly, it should support the immune system's efforts to eliminate any stray malignant cells. *Astragalus membranaceus* (astragalus root) is a common Chinese herbal medicine (CHM) with well-documented immunomodulating properties. Cho and Leung^[7, 8]^ isolated a potent bioactive fraction from astragalus root that demonstrated varied immune stimulating actions, both *in vitro* and *in vivo*. Specifically, macrophage number and phagocytic activity were increased, interleukin-2 expression was enhanced and tumors in murine models were suppressed. Other studies also revealed that a decoction containing astragalus root and *Angelica sinensis* (Chinese angelica root) induced secretion of interleukin-2^[9]^ and astragalus root alone was found to regulate macrophage immune responses^[10]^.

A Cochrane review has found 4 studies using astragalus root species to treat chemotherapy side effects in colorectal cancer patients. These studies showed that astragalus root administration significantly reduced the frequency of nausea and vomiting due to chemotherapy, reduced the incidence of clinically significant leucopenia, enhanced T-cell lymphocyte counts for certain subsets (CD3, CD4 and CD8) and did not affect immunoglobulin levels. However, these studies were of low methodological quality^[11]^.

### Improving prognosis: increase potency of chemotherapy

In addition to help patients minimize toxic side effects of chemotherapy and maximize their quality of life (QoL), integrating Chinese and Western medicine may also improve survival outcomes. Some evidence-based medicine reviews found the potential efficacy of CHM in treating hepatocellular carcinoma. A meta-analysis of 26 studies (2 079 patients) showed a statistically significant increase in survival for those who used CHM plus chemotherapy compared to those who used chemotherapy alone. However, the trials were of low quality and confirmation with large, quality studies is needed^[12]^. Most recently, a meta-analysis of CHM in combination with arterial chemoembolization of hepatocellular carcinoma found that addition of CHM to chemotherapy resulted in higher leukocyte (including T-cell and natural killer cell) levels with lower α-fetoprotein levels, reduced side effects of conventional therapy, improved the patients' QoL and also prolonged their survival. However, limited data and heterogeneity of the studies examined made definitive recommendations difficult, with further trials needed^[13]^.

On the other hand, result of a *meta*-analysis of 34 studies using astragalus root combined with platinum-based chemotherapy for advanced non-small cell lung cancer suggested that the combination might be more efficacious than chemotherapy alone. An extensive literature search revealed 1 305 relevant publications, of which 34 studies (2 815 patients) met the inclusion criteria. Thirty studies (2 472 patients) revealed improved tumor response and 12 studies (940 patients) showed increased survival at one year. The authors concluded that astragalus-based CHM may increase the effectiveness of platinum-based chemotherapy, but they noted that confirmation with rigorously controlled studies is warranted^[14]^.

There were also several studies investigating the use of CHM for nasopharyngeal carcinoma. In a meta-analysis of CHM plus conventional therapy versus conventional therapy alone for nasopharyngeal carcinoma, 18 RCTs (1 732 patients) met the inclusion criteria and six of them reported improved tumor response in the combination group^[15]^.

## Supportive cancer care with acupuncture

### Acupuncture treatment for pain

Acupuncture was first known to the conventional medicine world by its demonstrated analgesic property. Subsequent studies have suggested possible mechanisms through induced endorphin secretion and modification of thalamus and cortical activities in functional magnetic resonance imaging studies^[16, 17]^. Intraoperative use of acupuncture and related techniques has been examined in a few RCTs. In one trial, patients undergoing hip arthroplasty were randomized to auricular acupuncture and sham control. The treatment group was treated with indwelling needles to lung, shenman, forehead and hip points while the control group received needles to four non-acupuncture points on the helix. The results showed a reduction of 21% of fentanyl during surgery in the treatment group^[18]^. Several other RCTs also supported the effect of auricular acupuncture on anaesthetic requirements^[19, 20]^. However, acupuncture on a few selected body acupuncture points was not shown to be effective in reducing anaesthetic requirement^[21]^.

Although acupuncture analgesia has been studied in the laboratory and clinic for several decades, few acupuncture clinical trials exist for cancer-specific pain. In a single-blind RCT, 90 patients with cancer pain despite stable analgesic treatment were divided into three groups; one group received two courses of auricular acupuncture at points where an electrodermal signal had been detected, one group received auricular acupuncture at points with no electrodermal signal (placebo points) and the remaining group received auricular seeds fixed at placebo points. Treatment efficacy was based on the absolute decrease in pain intensity using the visual analog score measured two months after randomization. At two months, pain intensity had decreased by 36% from baseline in the group receiving acupuncture; there was little change for patients receiving placebo (2%). The difference between groups was statistically significant. This study represented a clear benefit from auricular acupuncture for cancer patients with ongoing pain despite analgesic therapy^[22]^.

Evaluation of acupuncture effect in acute postoperative pain control is hampered by problems of appropriate sham control, placebo effects and multiple confounding variables. With the increase in evidence from RCTs demonstrating the effectiveness of acupuncture and related techniques in postoperative pain control, acupuncture will likely be continuously used and examined as a component of acute pain control strategies after cancer surgery^[23]^. The types of acupuncture techniques to be utilized should be carefully chosen to balance the ease of delivery and expected effectiveness based on TCM principles and practice.

### Acupuncture treatment for nausea and vomiting

Postoperative nausea and vomiting is common among cancer patients following anaesthesia and surgery. A variety of acupuncture and related techniques have been evaluated for its effectiveness in the perioperative period for reduction of postoperative nausea and vomiting. Two studies showed that acupuncture treatment at acupoint Neiguan (PC6) could increase the anti-emetic effect of drugs for perioperative and chemotherapy-induced nausea and vomiting^[24, 25]^. Another RCT of 142 cancer patients demonstrated that acupuncture (20 min, once every other day for 20 days) plus point injection of vitamin B6, which is commonly used to relieve nausea and vomiting, at PC6 (50 mg each side) significantly decreased episodes of emesis and increased the number of emesis-free days compared to acupuncture or vitamin B6 (50 mg, twice a day for 21 days) alone. The results suggested that this combination might be useful against emesis in cancer patients^[26]^. Stimulation of PC6 may be done more conveniently with a small transcutaneous nerve stimulation device, such as the Reliefband, which is worn like a wrist watch.

In addition to PC6, Zhigou (TE6) and Zusanli (ST36) are also considered to be appropriate acupoints for treatment of vomiting and nausea induced by radiotherapy and chemotherapy. A number of RCTs have confirmed the efficacy of acupuncture on vomiting and nausea, which led to the National Institute of Health (US) consensus statement that "acupuncture is a proven effective treatment modality for nausea and vomiting"^[27-30]^.

### Supportive cancer care with Qigong

Cancer patients receiving chemotherapy develop common side effects including oral mucositis; gastrointestinal upset with diarrhoea, nausea and vomiting; myelosuppression with lowered blood counts resulting in anaemia, bleeding and increased risk of infection; skin toxicities with hair loss and dermatitis; poor appetite with weight loss and general fatigue and poor QoL. From TCM perspective, most chemotherapies cause disturbance in the balance of the body-mind network, affect the vital energy (qi) of the spleen and kidney system resulting in syndrome patterns such as deficient spleen qi that manifests as diarrhoea; heart fire manifests as stomatitis; disturbed spleen and stomach qi with nausea and vomiting and physically as damage to the stomach and intestinal lining^[31]^. With weakening of the whole body-mind network, a reduction in the healthy qi is resulted with suppression of the immune system and a deterioration of the general status of patients.

A recent systematic review showed that the value of Qigong for supportive cancer care had not been adequately investigated. There were no large-scale rigorous clinical trials which could provide a definitive answer. Six RCTs tested the effects of qigong as supportive cancer care compared with usual care or herbal medicine and showed no significant differences in most outcome measures^[32-37]^, whereas 5 other controlled clinical trials showed favorable effects of Qigong^[38-42]^. Two RCTs suggested the effectiveness in prolonging life of cancer patients but one trail failed to do so^[35, 36]^. Since it is methodologically challenging to design rigorous trials for Qigong, most of the trials have a high risk of bias. In controlled clinical trials, the nature of the control intervention deserves consideration. A placebo for Qigong, effectively, does not exist. An absence of adequate statistical analysis of the variability of therapeutic protocols and poor quality of reporting are frequent methodological problems. The current evidence from RCTs on Qigong as supportive cancer care is not convincing, as the number of trials and the total sample size are too small to draw firm conclusions^[43]^.

## Conclusion

Further well-designed RCTs of CHM, acupuncture and Qigong with or without conventional therapy in cancer patients are needed to provide definitive scientific evidence to determine the optimal doses, duration and timing of their interventions that will optimize cancer patients' immunologic function, reduce tumor burden, improve QoL and prolong survival while minimizing the side effects (such as anorexia, fatigue, nausea and vomiting) of major conventional treatments^[44, 45]^.
